# Virological perspectives of the current situation of oncogenic viruses in Egypt: a review

**DOI:** 10.1186/s13027-025-00706-7

**Published:** 2025-11-14

**Authors:** Alaa H. Ibrahim, Ayman S. El-Habbaa, Ehab M. El-Nahas

**Affiliations:** Department of Virology, Faculty of Veterinary Medicine, Benha University, Po Box: 13736, Moshtohor, Toukh, Egypt

**Keywords:** ALV, Animal, BLV, Cancer, Egypt, Human, MDV, Oncogenic viruses, REV

## Abstract

Oncogenic viruses play a pernicious role in the development of cancer, causing various tumors in humans and animals. These viruses are of public health importance in developing nations. Worldwide, about 15–20% of cancer cases are related to viral infections. About 12% of all cancers in humans are attributed to oncoviruses. However, the accurate rate of cancers attributed to oncoviruses across all animal cancers remains uncertain. In some species, such as chickens and cats, oncoviruses are responsible for approximately 80–100% of specific cancer cases. The first oncovirus reported in humans was the Epstein-Barr virus, in the case of Burkitt’s lymphoma. The Jaagsiekte sheep retrovirus was the first oncovirus to be reported in livestock. The main reported tumor viruses of veterinary importance are bovine leukemia virus, jaagsiekte sheep retrovirus, feline leukemia virus, bovine papillomavirus, equine papillomavirus, Marek’s disease virus, avian leukosis virus, and reticuloendotheliosis virus. The control of oncoviruses mainly relies on early molecular methods, such as PCR, with remaining difficulties concerning virus isolation. Moreover, the potential for viral oncogenes to integrate into host genomes underscores the challenges in diagnosis, control, and eradication. These viruses have a significant impact on veterinary health in Egypt, affecting various animal species and posing substantial economic challenges. Therefore, exploring the up-to-date situation of oncogenic viruses recorded in Egypt is essential. This review aims to elucidate the general mechanisms of viral oncogenesis, shedding light on the situation of oncoviruses of veterinary importance that circulate in Egypt, as well as their diagnosis and control.

## Background

Oncogenic viruses cause different tumors in animals and humans in Egypt [1]. These viruses are either DNA viruses, such as papillomaviruses, herpesviruses, and hepadnaviruses, or RNA viruses, including retroviruses and flaviviruses [1, 2]. Oncoviruses are thought to be responsible for about 15–20% of all cancers in humans [3]. These viruses have a significant public health concern, particularly in developing nations, disadvantaged communities, and among individuals with weakened immune systems [4]. Recent studies have shown that about 23% of all cancers in humans are caused by infectious agents, including oncoviruses, bacteria e.g., *Helicobacter pylori*, and parasites e.g., *Schistosoma haematobium*.

Oncogenic viruses are established threats to human, animal health and productivity in Egypt, necessitating further research, improved diagnostics, and effective control strategies. These viruses remain a significant contributor to significant economic losses in Egypt, especially in the poultry sector. These losses occur primarily due to mortalities, carcass condemnations, and immune suppression, which enhances other opportunistic pathogens [5]. These viruses include avian leukosis virus (ALV), reticuloendotheliosis virus (REV), and Marek’s disease virus (MDV) [6]. Making a differential diagnosis based on histopathological lesions appears to be challenging [7].

ALV is an oncogenic *Alpharetrovirus* that causes a high mortality rate in addition to tumors and decreased fertility, leading to severe economic losses in the poultry sector worldwide [8]. Among all identified subgroups of ALV, subgroup J is regarded as the most prevalent [9]. In Egypt, among all subgroups, only subgroups A and J were detected in Egyptian poultry farms. On the other hand, REV is a *Gammaretrovirus*, primarily linked to immunosuppression, runting-stunting syndrome, visceral lymphomas, and thymus and bursal atrophy in Egyptian poultry flocks [10]. Recent studies in Egypt revealed the insertion of REVs LTRs in the MDV and *Avipoxvirus* field isolates.

MDV is an *Alphaherpesvirus* that induces tumors in various avian species [11]. It causes the transformation of T-lymphocytes, resulting in cutaneous and visceral tumors, immunosuppression, as well as neurological and ocular lesions [12]. MDV can be subdivided into three distinct serotypes: serotype 1 (Gallid herpesvirus 2), serotype 2 (Gallid herpesvirus 3), and serotype 3 (Meleagrid herpesvirus 1). However, out of the three identified serotypes, serotype 1 is the only one that causes tumors in chickens [13]. It is considered one of the most prevalent oncoviruses affecting the poultry sector in Egypt.

Bovine leukemia virus (BLV), also known as enzootic bovine leukosis (EBL), is a *Deltaretrovirus* that is recognized as the most prevalent neoplastic disease in cattle [14]. Once an animal is infected, it becomes seropositive after three weeks and maintains a long-lasting, persistent infection [15]. Some studies have linked BLV infection and the development of breast cancer in Egyptian women [16, 17]. Among the identified 12 genotypes of BLV, only two genotypes have been identified circulating in Egyptian dairy cattle: genotypes 1 and 4.

Jaagsiekte sheep retrovirus (JSRV) is a *Betaretrovirus* that affects the sheep population. It is also referred to as ovine pulmonary adenomatosis virus (OPAV), which is associated with neoplasia in type II pneumocytes, primarily in sheep and occasionally goats [18]. Natural infection with JSRV was first confirmed in Egyptian sheep in 2011 [19]. Further research is required to determine the prevalence and impact of JSRV on Egyptian local sheep breeds.

Feline leukemia virus (FeLV) is an oncogenic, immunosuppressive *Gammaretrovirus* that is globally distributed in domestic and small wild cats, causing lymphosarcoma [20, 21]. Papillomaviruses (PVs) are recognized as one of the oncoviruses that lead to benign tumors (warts) in humans, animals, and birds [22]. Bovine papillomavirus (BPV) was reported in Egypt, while Equus caballus papillomavirus (EcPV) requires further research to reveal its impact on local breeds of horses in Egypt.

This review follows a narrative approach to provide a comprehensive overview of oncogenic viruses in Egypt, highlighting the situation of oncoviruses of veterinary importance, including their epidemiological findings, detection, diagnostic methods, and control strategies based on available peer-reviewed literature in major databases.

## General mechanisms of viral oncogenesis

**Fig. 1:**
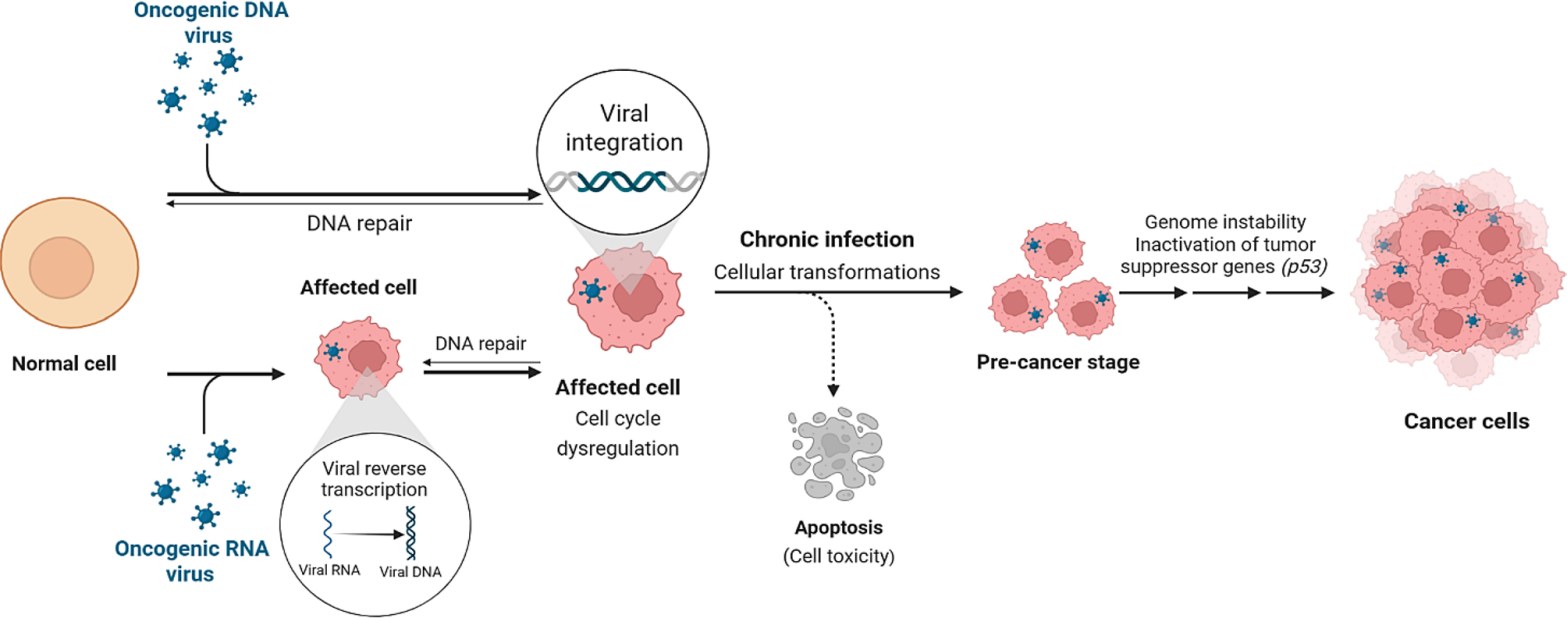
A diagram illustrates simply the mechanism of viral oncogenesis and cellular transformation. Following the infection of a normal cell by an oncogenic virus, the viral DNA integrates into the host genome, leading to cell cycle dysregulation and establishment of chronic infection. If DNA repair mechanisms fail, this can drive the cell through a pre-cancerous stage, often involving the inactivation of tumor suppressor genes (*p53*), eventually leading to cancer cell formation. Alternatively, viral cytotoxicity may trigger apoptosis of the infected cell. Original figure created by the authors using BioRender

Oncogenesis is a complicated process that comprises multistep events transforming a normal cell into a tumor one, as shown in Fig. 1. Most oncoviruses encode for oncoproteins that target the normal cellular proteins, like tumor suppressor gene *(p53)* which control cell apoptosis, and retinoblastoma *(pRb)* which play an essential role in shutting down the tumor suppression leading to the development of cancer [23]. Cellular transformation can be divided into three distinct steps: initiation, promotion, and progression [4]. Viral oncogenesis occurs due to the involvement of viral oncogenes *(v-onc)* that activate cellular proto-oncogenes *(c-onc)*, resulting in cell transformation, cell cycle dysregulation, and inactivation of tumor suppressor genes [24]. Therefore, the different mechanisms of viral oncogenesis were essential to be illustrated below, including the presence of viral oncogenes, cellular transformations, cell cycle dysregulation, and the inactivation of tumor suppressor genes.

### Presence of viral oncogenes (*v-onc*)

Viral oncogenes play an essential role in the indefinite growth of normal host cells and the synthesis of new viral gene-associated proteins, ultimately leading to cell transformation [25]. *V-onc* has its proto-oncogene analogs *(c-onc)*. Proto-oncogenes have a significant function in maintaining normal cellular growth. Once activation of the cellular oncogenes occurs, uncontrolled cell transformation develops [26]. The activation of proto-oncogenes requires genetic changes, which involve three mechanisms: mutation, gene amplification, and chromosomal translocations [27].

Some retroviruses, such as ALV, do not possess viral oncogenes, but can enhance cellular transformation by integrating their provirus alongside normal cellular oncogenes, leading to their expression through a process known as proviral insertional mutagenesis [28]. Oncogene products can be categorized into the following groups: growth factors, growth factor receptors, transcription factors, signal transducers, and apoptosis regulator factors [29]. It is now well established that all cellular and viral oncogenes in humans target normal cellular proteins and genomes to facilitate the progression of cancer [25].

### Cellular transformations

The integration of oncoviruses’ genetic material into the host cellular genome induces cellular transformations, mutations, and chromosomal translocations, leading to uncontrolled cell growth. Unlike DNA viruses, which integrate their genetic material directly into the host cell’s genome, RNA viruses require the reverse transcription of the RNA genome into DNA before integration into the cellular genome, disrupting cell metabolism and leading to cellular transformation [28].

### Cell cycle dysregulation

The cell cycle and homeostasis processes are known to be regulated by cyclin-dependent kinases *(CDKs)* and their inhibitors. Apoptosis is also considered a regulatory process that maintains the body’s homeostatic balance, and its dysregulation leads to the indefinite proliferation of cells [30]. Oncoviruses disrupt the homeostatic process through genetic mutations in the host genome, resulting in the continuous proliferation of infected cells [31].

### Inactivation of tumor suppressor genes

Tumor suppressor genes play a substantial role in protecting normal host cells from neoplastic transformations by regulating cell growth and division. Once tumor suppressor genes are inactivated by the interference of viral oncogenes, uncontrolled cellular proliferation occurs. The *p53* gene plays a vital role in inhibiting abnormal cell proliferation. Hepatitis B virus (HBV) encodes the hepatitis B X-oncoprotein *(HBx)*, which shuts down the *p53* gene, thereby blocking *p53*-mediated apoptosis [32].

## Oncogenic viruses in Egypt

Recent research revealed that oncogenic viruses are recognized as the leading cause for at least 20–25% of all cancers in both humans and animals in Egypt. Therefore, the up-to-date situation of these viruses in Egypt was briefly summarized in Table 1. The current situation of oncogenic viruses of veterinary importance that were reported in Egypt will be discussed below.

**Table 1:** The current situation of oncogenic viruses in Egypt

Families	Genera	Viruses	Species affected	Situation in Egypt	References
*Retroviridae*	*Alpharetrovirus*	Avian leukosis virus (ALV)	Avian	Reported	[33]
	*Gammaretrovirus*	Reticuloendotheliosis virus (REV)	Avian	Reported	[34]
	*Alpharetrovirus*	Avian sarcoma virus (ASV)	Avian	No data available	-
	*Alpharetrovirus*	Avian myeloblastosis virus (AMLV)	Avian	No data available	-
	*Deltaretrovirus*	Bovine leukemia virus (BLV)	Bovine	Reported	[35]
	*Betaretrovirus*	Jaagsiekte sheep retrovirus (JSRV)	Ovine	Reported	[19]
	*Gammaretrovirus*	Feline leukemia virus (FeLV)	Feline	Reported	[36]
	*Epsilonretrovirus*	Walleye dermal sarcoma virus (WDSV)	Fish	No data available	-
	*Deltaretrovirus*	Human T-lymphotropic virus type-1 (HTLV-1)	Human	Reported	[37]
*Herpesviridae*	*Mardivirus*	Marek’s disease virus (MDV)	Avian	Reported	[38]
	*Salmovirus*	Salmonid herpesvirus 2 (SalHV-2)	Fish	No data available	-
	*Lymphocryptovirus*	Epstein-Barr virus (EBV)	Human	Reported	[39]
*Papillomaviridae*	*Deltapapillomavirus*	Bovine papillomavirus (BPV)	Bovine	Reported	[40]
			Equine	Reported	[41]
	*Dyoiotapapillomavirus*	Equus caballus papillomavirus (EcPV)	Equine	No data available	-
	*Alphapapillomavirus*	Human papillomavirus (HPV)	Human	Reported	[42]
*Polyomaviridae*	*Alphapolyomavirus*	Merkel cell polyomavirus (MCPyV)	Human	Reported	[43]
*Hepadnaviridae*	*Orthohepadnavirus*	Hepatitis B virus (HBV)	Human	Reported	[44]
*Flaviviridae*	*Hepacivirus*	Hepatitis C virus (HCV)	Human	Reported	[45]

Note: “Not data available” refers to the absence of peer-reviewed publications documenting the presence of these viruses in Egypt, as indexed in major databases (e.g., Scopus, PubMed) up to the time of the review

## Veterinary oncogenic retroviruses in Egypt

Retroviruses infect various species of animals, including mammals, birds, fish, and reptiles, causing many economically important diseases [46]. All retroviruses are enveloped, single-stranded RNA-positive sense viruses [47]. They are about 80–100 nm in diameter with a unique genome comprising two identical diploid copies of positive-sense RNA [46]. The genome of retroviruses comprises four main genes that encode the virion proteins: *gag, pro, pol,* and *env.* The *gag* gene encodes the main structural polyproteins: matrix (MA), capsid (CA), and nucleocapsid (NC).

**Fig. 2:**
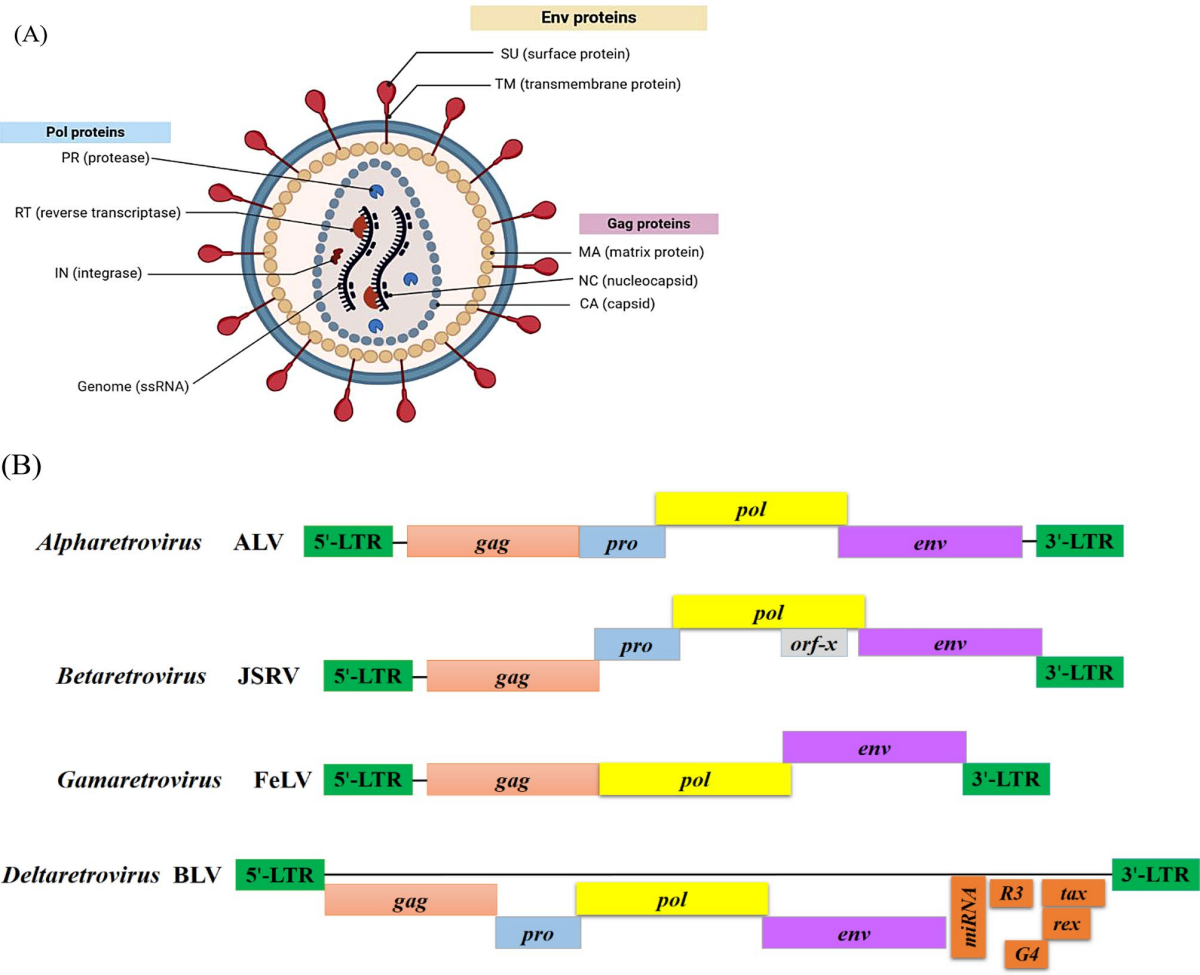
**A** Diagrammatic representation of the retrovirus virion, showing a single-stranded RNA genome, envelope proteins including SU and TM proteins, core proteins including MA, CA, and NC proteins, in addition to enzymes including PR, RT, and IN proteins. **B** Genome organization for different genera of retroviruses (*Alpharetrovirus*, *Betaretrovirus*, *Gammaretrovirus*, and *Deltaretrovirus*), showing the organization of core genes (*gag*, *pro*, *pol*, *env*) along with other accessory genes (*orf-x*, *tax*, *rex*, *R3*, *G4*, *miRNA*), and regulatory regions (5′-LTR and 3′-LTR). Original figure created by the authors using BioRender

The *pro* gene encodes a protease (PR), while the *pol* gene encodes a multifunctional protein that comprises the reverse transcriptase (RT) and integrase enzyme (IN). The *env* gene encodes two glycoproteins, an antigenic surface (SU) protein and a transmembrane (TM) protein, as shown in Fig. 2A. These viruses are uniquely characterized by containing a viral-coded reverse transcriptase enzyme, which transforms the genetic material from single-stranded RNA into double-stranded DNA in a process referred to as reverse transcription. After reverse transcription, they integrate themselves into the host cellular genome and are hence called proviruses [47].

The structural genes of retroviruses are organized as *(gag-pro-pol-env)*, and are flanked by two indistinguishable long terminal repeats (LTRs), as shown in Fig. 2B. This arrangement is almost conserved among all retroviruses [48], in addition to some accessory genes as in viruses of genus *Betaretrovirus* and *Deltaretrovirus*. *Retroviridae* is classified into two subfamilies: *Orthoretrovirinae* and *Spumaretrovirinae*. Orthoretroviruses are known to cause tumors and are formally referred to as oncoviruses. Unlike other retroviruses, Spumaretroviruses are non-oncogenic and do not cause tumors [46]. *Orthoretrovirinae* is further subclassified into six genera: *Alpharetrovirus*, *Betaretrovirus*, *Gammaretrovirus*, *Deltaretrovirus*, *Epsilonretrovirus*, and *Lentivirus* [46]. Oncogenic retroviruses of veterinary importance that were reported in Egypt include ALV, REV, BLV, JSRV, and FeLV. Therefore, it was essential to illustrate their situation in Egypt below.

### ALV

ALV is a contagious oncogenic *Alpharetrovirus* that infects various avian species, including layers, broiler chickens, ducks, as well as wild birds [49]. It is considered a high economic important inducing tumors, immunosuppression-associated infections, high mortality, retardation in growth, and a decline in egg size and production [50]. There are 11 recently identified subgroups (labelled A through K) based on the diversity of envelope surface glycoprotein *gp85*, the patterns of cross-neutralization, and host range.

Among these subgroups, only A, B, C, D, J, and K are exogenous and cause infection in chickens and turkeys [51]. On the other hand, the subgroup E strains are endogenous and recognized as non-pathogenic [52]. Subgroups F, G, H, and I are also recognized as endogenous and less commonly discussed. ALV-J is considered the most prevalent subgroup, causing multiple types of malignant tumors [9]. It is known that exogenous ALV subgroups are transmitted in chickens both vertically and horizontally, while endogenous subgroups are transmitted only vertically [53].

In Egypt, among the 11 identified subgroups of ALV, only subgroups A and J were detected in Egyptian poultry farms. Providing a historical perspective on the detection and emergence of ALV in Egypt is essential. It highlights the need for more epidemiological surveys, followed by an overview of the current situation of the virus. ALV-J was first detected in Egypt in 2000 in broilers, resembling the HPRS-103 strain [54]. From 2000 to 2004, several cases of ALV-J-infected breeder chicken farms were investigated [55]. Some cases of ALV-J were later detected in Egyptian flocks of broiler and layer chickens between 2000 and 2017 [56, 57]. Subsequently, ALV was the primary cause for avian neoplastic diseases in layer chickens in Lower Egypt during 2018–2019, revealing that 50% of tested tumor samples were positive for ALV-J [33].

ALV-J with myelocytomatosis was investigated in broilers in Sharqia, Dakahlia, and Qalyubia governorates, revealing that 67.5% of the tested flocks were seropositive for ALV-J [58]. ALV-J was also reported in Egyptian broiler chickens aged 28 days, based on histopathological findings and 26% seroprevalence [59]. Although ALV-J is rarely observed in ducks, it was detected in a breeder farm of commercial Peking ducks in Egypt in 2015 [60]. ALV-J was also molecularly detected by real-time RT-PCR in Egyptian duck farms in 2021 [49].

ALV-A has been diagnosed in Egypt in other avian species, rather than chickens. A recent study identified lymphoid leukosis in a breeder farm of pigeons in Ismailia governorate exhibiting tumors in various organs [61]. Therefore, recent studies indicated that ALV-J remains the most prevalent subgroup in Egypt, especially in key poultry-producing governorates, including El-Sharqia, El-Dakahlia, and Al-Qalyubia. Other subgroups may not be investigated due to limitations in surveillance.

Standard tools used for the diagnosis of ALV include PCR as a rapid and accurate assay, in addition to histopathological examination [62]. ELISA is also regarded as a proper and sensitive serological tool for screening ALV antibodies in breeder flocks [63]. Detection and characterization of ALV in Egypt involved different approaches, including serological screening by enzyme-linked immunosorbent assay (ELISA) [58], molecular identification by reverse transcriptase polymerase chain reaction (RT-PCR) targeting the *gp85* gene [33], and histopathological examination revealing the tumor with myeloid cells infiltrated with eosinophilic granular cytoplasm [49].

ALV can be isolated on the chicken embryo rough (CER) cell line; the cytopathic effect (CPE) is characterized by cell aggregation, rounding, and detachment [64]. Because most ALVs do not produce visible CPE on cell culture, several diagnostic tests are used for viral identification, such as the complement fixation test (CFT) and ELISA [65]. Concerning inoculation on specific pathogen-free (SPF)-ECEs, ALV can also be isolated on 9-day-old ECEs, then incubated at 37 °C for 5–7 days; the signs are shown as an enlarged liver with stunting, dwarfism, curling embryo, and congestion of the chorioallantoic membrane (CAM) [66].

Over the past few decades, vaccine contamination with ALV has been increasingly reported in the Egyptian market [67]. Therefore, RT-PCR and ELISA were performed to screen the commercial live vaccines of MDV, detecting cross-contamination with ALV [50, 68]. The complement fixation test for avian leukosis (COFAL) is a routine test used during vaccine manufacturing to detect any cross-contamination with ALVs [69]. Other regulatory actions may provide a solution, including strict quality control measures during vaccine production, routine screening for contaminants, and enhanced oversight by veterinary authorities.

Additionally, there is no commercially available vaccine for ALV. Hence, strict control strategies are employed in poultry farms to reduce the spread of viruses [33]. Additionally, it was recommended to eradicate the positive birds and monitor for the circulating Egyptian strains of ALV periodically [49]. These control strategies may include regular surveillance, genetic analysis, biosecurity measures, and culling of infected birds. Continuous research and collaboration among veterinary institutions are crucial for developing effective strategies to control ALV and mitigate its impact on Egypt’s poultry sector.

### REV

REV is a *Gammaretrovirus*, recognized as an oncogenic and immunosuppressive virus [70]. It affects different avian species, including chickens, ducks, geese, turkeys, and pheasants [71]. REV frequently occurs in aged-bird flocks, including layer and breeder chickens [72]. Mallard ducks were known to resist the REV infection until it was first isolated from mallards in China [73].

REV is unrelated to the ALS group either immunologically, morphologically, or structurally [74], but is more closely related to mammalian retroviruses [75]. There is only one serotype of REV [76]. It was recognized to have a tropism in kidneys, lymphoid organs, epithelial cells, and blood cells [77]. It is known to induce lymphomas in T-cells or B-cells; however, histopathology and lymphocyte markers cannot differentiate between lymphomas caused by REV and those induced by MDV and ALV [78].

In Egypt, numerous studies on REV diagnosis were performed [79–81]. REV was detected in Egyptian broiler breeder flocks with visceral tumors in 2005 in the Giza governorate [82], relying on PCR, ELISA, and histopathological findings. REV was first reported in cross-bred commercial chickens in the delta region during 2011–2012 [83]. REV status was monitored by PCR and ELISA in 39 commercial chicken farms across 11 Egyptian governorates between 2019 and 2021, revealing that six farms were positive in three governorates: El-Sharqia, El-Minya, and El-Beheira [84].

Recent studies revealed the insertion of REVs LTRs in the MDV field isolates circulating in Egypt [85]. REV causes tumorigenic and immunosuppressive effects, with a seroprevalence rate reaching 35%, and has spread in three central poultry-producing governorates in Egypt, including Ismailia, Sharqia, and Dakahlia [34]. REV 5′ LTR was studied in the integrated form within the genome of *Avipoxvirus* field strains circulating in different bird species in Egypt [86]. The integration of REV into the genomes of MDV and fowl poxvirus (FPV) field isolates may alter the pathogenicity and virulence of these viruses.

Diagnosis of REV mainly depends on ELISA, which is considered a more appropriate sensitive screening tool than the indirect immunofluorescent antibody technique (IFAT) [87]. Additionally, PCR is an accurate and sensitive tool for diagnosing REV infections [34]. The diagnostic approaches for REV in Egypt mainly depend on serological screening using ELISA, molecular identification via RT-PCR [84], histopathological examination, and immunohistochemistry (IHC) [34]. REV can be propagated on avian-origin cell lines, such as chicken embryo fibroblasts (CEFs) or duck embryo fibroblasts (DEFs), producing a CPE of discrete, multiform syncytia [88].

Vaccine contamination with REV is recorded, particularly during the preparation of MDV and FPV using chicken cell culture [72]. Contaminated vaccines can inadvertently introduce REV into poultry populations, leading to outbreaks and reducing the effectiveness of vaccination programs. Unfortunately, there are currently no commercially available vaccines or specific medications for REV. Therefore, REV could be generally controlled by strict measures of biosecurity and culling of positive breeders [89]. Conducting intensive surveillance programs is essential for monitoring REV prevalence and genetic variations, as well as vaccine quality control.

### BLV

BLV is an oncogenic *Deltaretrovirus,* which is closely related to human T-cell leukemia virus types 1 and 2 (HTLV-1 and HTLV-2) [14]. It is considered the most common neoplastic disease affecting cattle worldwide [14]. It causes significant economic losses in cattle farms, either directly by reducing milk production and cow lifespan, or indirectly through restrictions on animal and product imports from endemic areas [90]. It naturally infects domestic cattle and is often seen in buffaloes and camels [91]. However, a recent study in Egypt confirmed that camels are resistant to BLV infection [92]. Genotyping of BLV based on the *gp51* gene identified 12 different genotypes [93].

Three genotypes are considered the most prevalent worldwide: genotypes 1, 4, and 6 [94]. Several recent reports have suggested a possible relationship between BLV and the development of breast cancer in women, in addition to other hematopoietic neoplastic diseases [95]. The transmission method of BLV to humans remains unrecognized; however, the consumption of infected raw milk can transmit the virus from cattle to humans [96].

Consequently, a recent study confirmed that there was a significant correlation between the *tax* gene of BLV and breast cancer development in admitted cases of women in Egypt [16, 17], which is known to cause about 33% of cancer-related deaths [97]. The previous studies suggest such a correlation based on the detection of the integrated proviral DNA in breast tumor tissues. However, a direct causal relationship between them remains unconfirmed. Despite some traditional habits in certain Egyptian rural areas, consuming raw milk without sterilization or pasteurization, as well as direct contact with animals under low hygienic conditions.

In Egypt, only two genotypes have been identified circulating in dairy cattle: genotypes 1 and 4 [98]. Another study revealed the genotype-1 of BLV in a blood sample collected from cattle in Egypt [35]. It was first identified in Egypt in 1996, in Assiut, Arab El-Aoumar, with a seroprevalence rate of 37.7% in imported dairy cows under two years old and 72.8% in cows above two years old [99]. Although Egypt has been officially listed as BLV-free since 1997 by the World Organization for Animal Health (formerly OIE) [100], several recent studies have reported BLV seropositivity and proviral DNA detection in Egyptian cattle populations, suggesting underreporting or a lack of updated international reporting systems.

A survey of BLV reported an overall seroprevalence of 18.2% among Egyptian dairy cattle, with the highest prevalence in Kafr El-Sheikh province (28.4%) and the lowest prevalence in Gharbia province (7.1%) [35]. BLV was first reported among beef cattle in Egypt in 2018 [101]. Egyptian grazing cattle with a loose housing system are more susceptible to the BLV infection [35]. The significant factors contributing to the spread of BLV among Egyptian cattle include the importation of contaminated frozen semen carrying BLV and the introduction of unscreened heifers for the virus [102].

BLV could be isolated on fetal bovine lung (FBL) cells, with subsequent growth for 3–4 days, with development of CPE as syncytia in the monolayer cells [100]. ELISA is a rapid diagnostic tool used for screening cattle against BLV [103]. PCR was also proposed to be involved in the routine laboratory tests used for breeding cattle for international trade [104]. Diagnostic techniques for BLV in Egypt mainly depend on seroprevalence testing using ELISA [105], the agar gel immunodiffusion test (AGID), and PCR [106]. PCR was recommended for BLV diagnosis [106], due to its high sensitivity and considered more economical compared to commercial ELISA and AGID.

Egypt has not yet developed strict measures for control of BLV infection in dairy cattle farms [98]. The established control measures in most European countries include testing, segregation, and trials to develop a novel vaccine [91]. Therefore, it is recommended to import certified dairy cows from countries free from BLV infection. Besides, the screening of serum and milk for BLV-specific antibodies is a good indicator of the disease [99]. BLV infection in Egyptian cattle herds can be mitigated through recommended control measures, such as strict biosecurity, regular seroprevalence, and genetic selection of breeds.

### JSRV

JSRV is a *Betaretrovirus*, also known as OPAV, which causes contagious chronic tumors in sheep but less commonly in goats [107]. It mainly affects adult animals, causing progressive respiratory disease. It has a high economic importance, due to lambs’ mortality, retarded growth, and carcass condemnations in abattoirs [108]. JSRV is the leading cause of about 70% of lung tumors in sheep [109]. It was first reported through genome sequencing in the 19^th^ century in South Africa, where it was referred to as jaagsiekte, meaning “chasing sickness” in Afrikaans [110].

JSRV-infected sheep show dyspnea, anorexia, emaciation, and signs of pneumonia with no response to antibiotic treatment, and death within a few weeks [111]. JSRV histopathological findings are similar to bronchioloalveolar carcinoma (BAC) in humans [112]. Gross lesions vary from multifocal greyish nodules to complete consolidation of the affected lung [108] and frothy exudate in the tracheal passages. A clear demarcation is observed between the tumor tissue and normal rosy tissue on the lung incision [113]. JSRV-infected pneumocytes are characterized histopathologically by a papillary or acinar adenocarcinoma, surrounded by connective tissue infiltrated with mononuclear inflammatory cells [113].

In Egypt, only a few studies have been performed on JSRV in sheep farms. It was not confirmed until the first confirmation of natural infection was described in Egypt during 2008–2009 [19]. The study was conducted on slaughtered sheep in Beheira Province from August 2008 to September 2009. In which JSRV-CA protein was demonstrated in the lungs of 7 sheep among 550 by IHC analysis, confirming the results by histopathological examination. In addition, a case report described the histopathological findings of a small JSRV outbreak on an Egyptian sheep farm [114]. Phylogenetic analysis showed that the detected JSRV strains have 100% similarity with known reference strains, indicating the circulation of established viral lineages in Egypt [115]. JSRV has limited recorded data in Egypt, so further research is necessary to determine its prevalence in sheep farms.

Unfortunately, commercial vaccines and treatments for JSRV are not yet available. Therefore, the most effective control measures include quarantine, disinfection of contaminated farms, and culling infected animals [109]. Early detection of JSRV infection is challenging due to limitations in preclinical diagnosis. Histopathology, IHC, and PCR targeting the LTR region are recommended methods for diagnosing JSRV [116]. Additionally, strict biosecurity measures are essential, including mandatory screening and culling of positive sheep cases [117].

### FeLV

FeLV is an oncogenic, immunosuppressive *Gammaretrovirus* affecting domestic and small wild cats worldwide [21]. FeLV was first reported in Scotland in 1964 by William Jarrett and his coworkers, isolated from a cat with natural lymphosarcoma [118]. FeLV infection in domestic cats is considered a significant cause of death due to its ability to cause bone marrow disorders, immune suppression, and hematopoietic neoplasia with the progressive form of the disease [20]. Its prevalence varies according to region; Europe has a prevalence rate reaching 8.8% [119], while Egypt has limited data on the prevalence of FeLV.

It is related to HTLV [36]. FeLV comprises three major subtypes: FeLV-A, FeLV-B, and FeLV-C. Each subtype utilizes a distinct host cellular receptor; besides, FeLV-B and FeLV-C are the most pathogenic subtypes [20]. Cats infected with FeLV show clinical signs, as fever, lethargy, lymphadenopathy, pale mucous membranes, and immune-mediated disorders [20]. In addition to bone marrow disorders, anemia, and cytopenia [120]. Vaccination of domestic cats against FeLV could reduce the prevalence of infection in many geographical areas [121]. The first commercial vaccine against FeLV was developed on the FL-74 cell line [122].

In Egypt, a seroprevalence study in Cairo found that only 8 of 174 feral cats tested were seropositive for FeLV, resulting in an incidence of 4.6% [36]. A molecular study would be necessary to identify the subtypes circulating in Egypt, as previous research has focused on seroprevalence studies only, without further subtyping. FeLV can be efficiently isolated using specific feline-derived cell lines, such as Crandell-Rees feline kidney (CRFK) cells or feline embryonic fibroblasts (FEFs) [21]. No distinct CPE typically appears, requiring specific assays to confirm FeLV [123], such as immunofluorescence assay (IFA), ELISA, and RT-PCR [124].

Diagnostic techniques for FeLV mainly rely on blood tests, serum biochemistry, urinalysis, radiography, and sonography [119]. The recommended control measures include regular screening, vaccination, keeping cats indoors, and isolating infected cats [119]. FeLV remains a significant threat to the health and welfare of cats in Egypt, with limited diagnostic options. Therefore, collaboration between veterinarians and animal welfare organizations is essential to improve the overall well-being of Egypt’s feline population.

## Veterinary oncogenic herpesviruses in Egypt

**Fig. 3:**
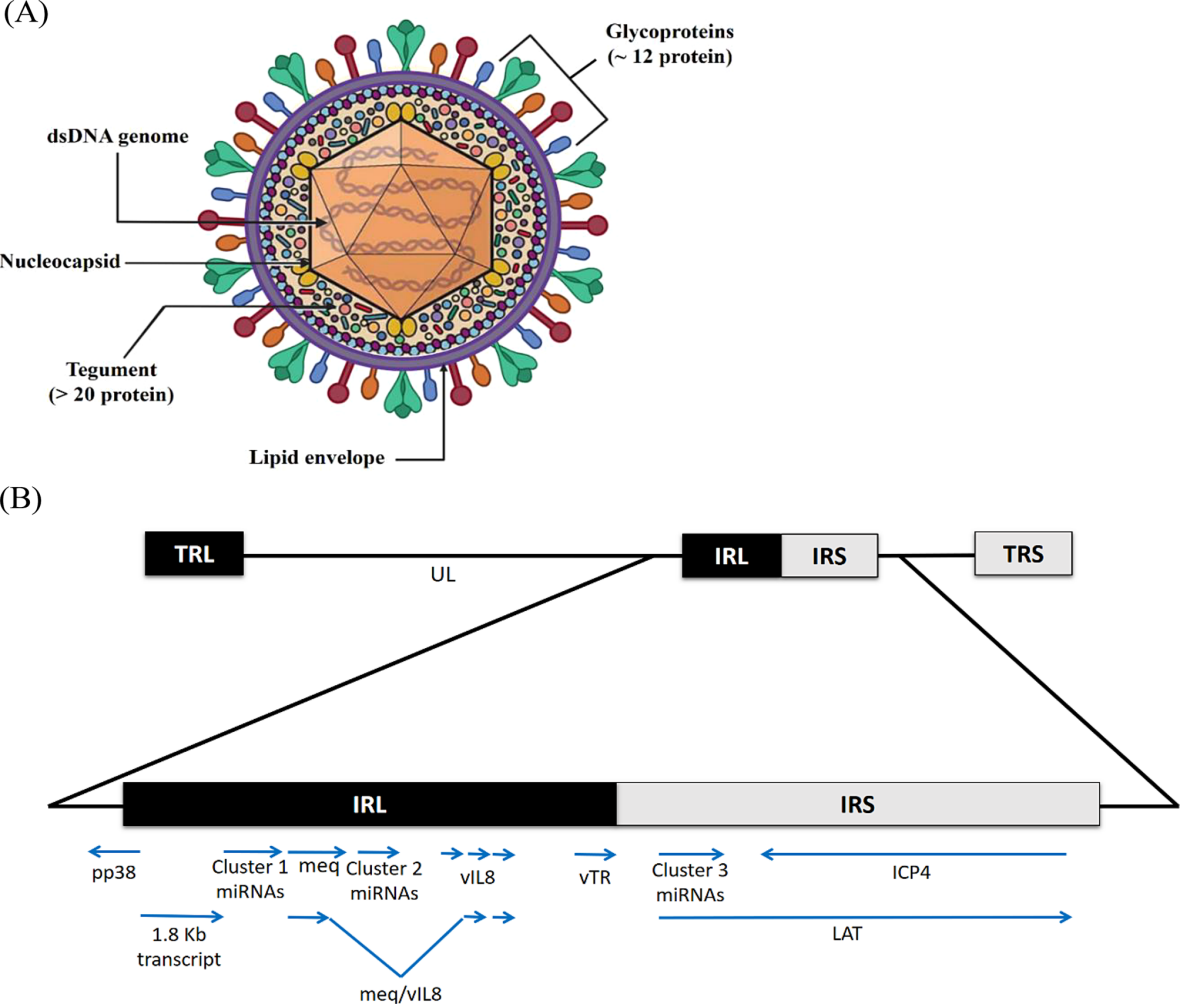
**A** Diagrammatic representation of the herpesvirus virion, highlighting its structural components: a double-stranded DNA genome enclosed in a nucleocapsid, surrounded by a protein-rich tegument layer, a lipid envelope, and surface glycoproteins. **B** Genome organization of MDV as an oncogenic *Alphaherpesvirus*, illustrating the complexity of gene regulation in oncogenic herpesvirus genes. Original figure created by the authors using BioRender

Herpesviruses infect many animal species, with a wide natural distribution [125]. Herpesvirus virions are enveloped with a double-stranded DNA genome which wrapped into a capsid of icosahedral symmetry, in addition to a protein layer which is loosely organized referred to as the tegument surrounds the viral capsid and glycoproteins comprise about 12 proteins, as shown in Fig. 3A. The tegument layer comprises many viral proteins that play an essential role in the infection of host cells and viral genes’ expression [126].

After the tegument layer and envelope are included, virion size becomes approximately 300 nm in diameter, with a genome varying from 120 to 250 kb, and encodes 70–220 open reading frames (ORFs) [127]. According to the 2024 ICTV taxonomy, the order *Herpesvirales* includes three families: *Herpesviridae*, *Alloherpesviridae*, and *Malacoherpesviridae* [128]. The *Herpesviridae* family is classified into three distinct subfamilies: *Alphaherpesvirinae*, *Betaherpesvirinae*, and *Gammaherpesvirinae*. Alphaherpesviruses have a broad host range and establish a latent infection in sensory neuronal cells. Furthermore, they are subclassified into four genera: *Simplexvirus, Mardivirus, Iltovirus,* and *Varicellovirus* [129].

Among alphaherpesviruses, *Gallid alphaherpesvirus 2* (GaHV-2) is a highly contagious and spreading virus that belongs to the genus *Mardivirus*, which causes Marek’s disease in chickens, and has been reported in Egypt [38]. Otherwise, betaherpesviruses have a narrow host range [127], establishing a latent infection mainly in macrophages and monocytes. Furthermore, they are subclassified into four genera: *Cytomegalovirus*, *Roseolovirus*, Muromegalovirus, and *Proboscivirus* [130].

Unlike alphaherpesviruses, gammaherpesviruses have a narrow host range, establishing latency in either T or B-lymphocytes [131]. Furthermore, they are subclassified into four genera: *Lymphocryptovirus*, *Macavirus*, *Rhadinovirus*, and *Percavirus* [132]. MDV is recognized as the most significant oncogenic herpesvirus affecting the poultry sector. Therefore, it was essential to illustrate its situation in Egypt below.

### MDV

MDV is a serious avian *Alphaherpesvirus* that affects chickens and, occasionally, turkeys. In the global poultry industry, it is considered one of the most economically devastating contagious diseases [132]. It causes significant mortalities, neuro-lymphoproliferative, and immunosuppressive disorders in chickens [133]. It was first described in cockerels with polyneuritis in Hungary in 1907 [134]. MDV has a genome encoding more than 200 genes [135]. The most important genes related to the virulence and oncogenicity include the Marek’s EcoRI-Q *(Meq)* gene and the infectious cell protein-4 *(ICP4)* gene, as shown in Fig. 3B.

The mutation in the *Meq* gene has been associated with the determination of viral virulence, oncogenicity, and genetic diversity [136]. Therefore, deletion of the *Meq* gene results in the loss of MDV oncogenicity, as described in MDV serotypes 2 and 3 [137]. The *ICP4* gene is a homologous gene within alphaherpesviruses, which is grouped under the main category of MDV genes [138]. MDV is subdivided into three serotypes, which differ in pathogenicity and virulence [139]. MDV serotype 1 (CVI988) is considered the only serotype causing disease in chickens; otherwise, serotypes 2 (SB-1) [140] and 3 (turkey herpesvirus; HVT) are naturally avirulent, non-pathogenic, and non-oncogenic strains which are used as vaccinal strains for flocks immunization [139].

Oncogenic serotype 1 differs from other serotypes due to the presence of the *Meq* oncogene and several unique genes, including *pp38*, *vIL-8*, and *vTR*, in repeat regions [141]. Additionally, MDV serotype 1 is further classified into four pathotypes based on virulence, as follows: mild (m), virulent (v), very virulent (vv), and very virulent plus (vv+) [142]. Recently, PCR has been demonstrated to be a serotype identification tool [143], and it is also used to differentiate between vaccinal and field strains of MDV-1 [144]. Clinical signs of MDV include peripheral nerve enlargement resulting in leg and wing paralysis, and cloudy eyes. In addition, solid tumor masses in visceral organs, such as the liver, spleen, and ovaries, originate from transformed T-lymphocytes [145, 146].

In Egypt, the classical form of MDV was first reported in 1954 [147]. MDV was first isolated from Egypt at the beginning of this century [148]. Hence, he had isolated three highly virulent MDV strains from the buffy coat layer of 25 healthy broiler flocks. According to the OIE, Egypt has recently been identified as an endemic area for MDV infections, with outbreaks reported both before and after 2009 [149]. MDV-3 (HVT) vaccines cannot provide complete protection against virulent strains of MDV [150]. Thus, it was recommended to perform the bivalent vaccination strategy in Egyptian broiler breeder flocks to induce protection against the infection [151].

A previous Egyptian study aimed to isolate MDV from infected chicken flocks on ECEs via the CAM route, and viral identification was performed using serological tests such as the agar gel precipitation test (AGPT) and the indirect fluorescence antibody technique (IFAT), supported by molecular identification using conventional and real-time PCR [152]. Egyptian poultry flocks continue to suffer from recurrent outbreaks of MDV, primarily due to increased viral virulence [138]. The evolution of MDV strains leads to an increase in virulence, highlighting the need for intensive surveillance, updated vaccination strategies, and improved farm-level practices to control the virus in Egypt.

MDV was screened in samples from Egyptian layer farms across various localities in Egypt. Such isolates were characterized by sequencing of the *ICP4* gene to differentiate between field and vaccine strains [138]. The prevalence of MDV was described during the 2020 outbreak in flocks in Lower Egypt, with genetic characterization of the *Meq*, *gL*, and *ICP4* genes in field isolates [153]. The Egyptian strains of MDV should be genetically related to those circulating in Ethiopia, China, and India [145]. MDV was first identified in turkeys in Fayoum and Minya governorates in 2018, marking the first time it was reported among turkeys in Africa and the Middle East [38].

Phylogenetic characterization of Egyptian strains of MDV showed similarities with those of Ethiopia, India, and China, suggesting possible international transmission routes [145]. Serotype 1 (vv+) of MDV was identified in commercial layers aged 16–40 weeks during 2015–2019 in eight Egyptian Governorates, Qalyubia, Sharqia, Dakahlia, Gharbia, Beheira, Alexandria, Giza, and Fayoum [146]. REV-LTR insertions were first identified and characterized in Egyptian MDV field isolates in a study conducted from 2016 to 2018 [145]. Such insertions may lead to more severe immunosuppression, tumor development, and complicated control efforts.

MDV could be isolated on cultures of chicken kidney (CK) cells, CEF, and DEF. With subsequent growth for 3–4 days [138], with CPE as cell clumping and aggregation [154]. Diagnosis of MDV in Egypt primarily relies on clinical findings, gross pathology, serological testing by AGPT and IFAT [152], histopathological examination [146], and molecular detection by PCR targeting the *ICP4* gene [138].

Although the intensive CVI988 vaccination strategy, MDV outbreaks are still documented in both vaccinated and non-vaccinated chicken flocks worldwide. This may be attributed to the increased virulence of the virus [139]. MDV remains a significant threat to the poultry industry in Egypt. Therefore, it is essential to establish regular surveillance, including the molecular characterization of circulating strains, vaccine safety and evaluation, genetic selection of breeds, and strict biosecurity measures, in high-poultry-producing governorates.

## Veterinary papillomaviruses in Egypt

PVs are oncoviruses that cause benign tumors or warts in humans, animals, and birds. PVs induce mucosal and epitheliotropic tumors, which carry a significant risk of malignant progression [22]. Furthermore, previous studies have found that HPVs are a substantial cause of cervical cancer in humans [155]. PV infections are implicated in about 5% of all human cancers [156]. PVs were previously grouped with polyomaviruses in one family, known as *Papovaviridae*. According to the current classification by the International Committee on the Taxonomy of Viruses (ICTV), they are classified as two separate families: *Papillomaviridae* and *Polyomaviridae* [22]. PVs now belong to the family *Papillomaviridae* [157].

**Fig. 4:**
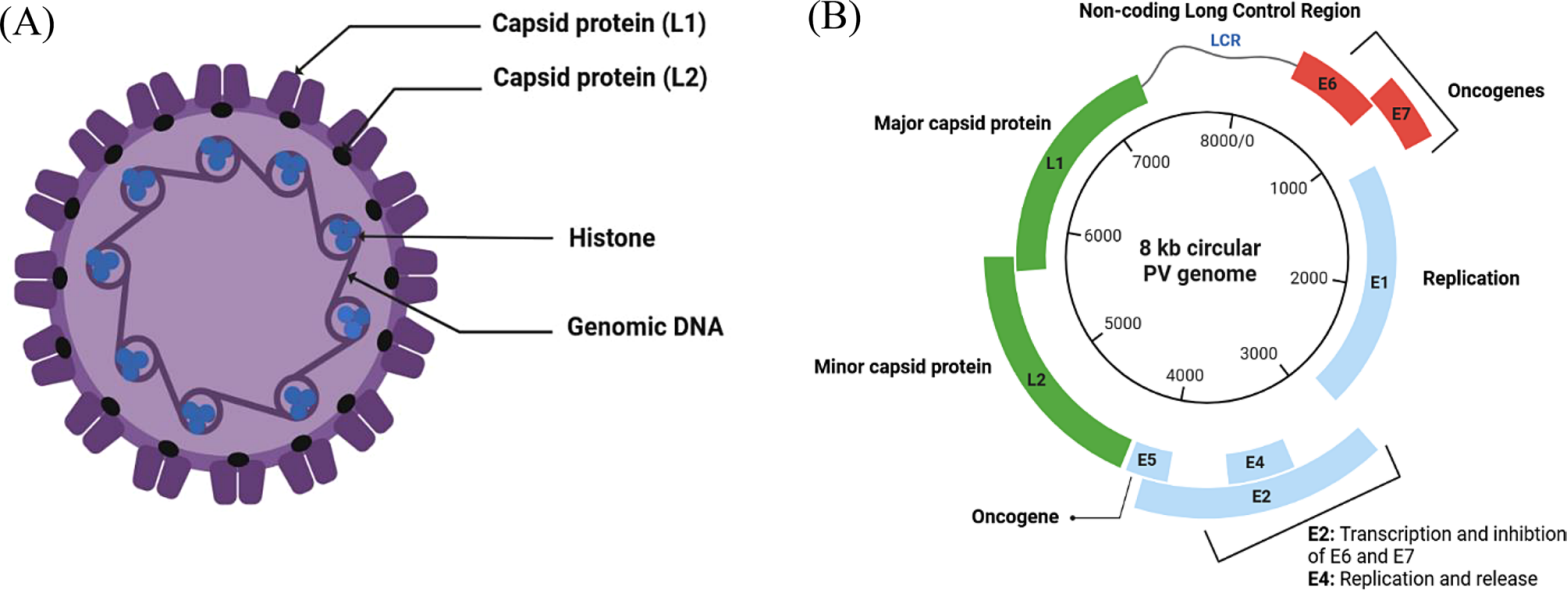
**A** Diagrammatic representation of the papillomavirus virion, highlighting the capsid proteins (L1 and L2), histones, and circular double-stranded DNA genome. **B** Genome organization of the papillomavirus, illustrating the function of significant genes, including oncogenes (E6, E7), replication-associated genes (E1, E2, E4, E5), capsid proteins (L1, L2), and the non-coding long control region (LCR). Original figure created by the authors using BioRender

PVs are non-enveloped viruses with a circular, double-stranded DNA genome associated with histones, as shown in Fig. 4A, which is approximately 8000 base pairs (bp) in size. The PV genome encodes about 10 proteins, including the major capsid protein (L1) and the minor capsid protein (L2), which comprises the capsid. The remaining proteins are non-structural early proteins, labeled as E1 through E8, as shown in Fig. 4B. PVs can be classified based on the highly conserved L1. If the L1 ORF of one PV shows less than 90% similarity to that of other PVs, it is considered different [158]. In brief, the PV genome consists of three oncogenes: E5, E6, and E7, which induce transformation. Additionally, two regulatory genes, E1 and E2, modulate both transcription and replication processes, while the two structural proteins, L1 and L2, form the viral capsid [159].

E1, E2, L1, and L2 are identified as highly conserved among all PVs [22]. PV infection may be associated with immunodeficiency as a risk factor, as PV infection has been documented in domestic cats infected with feline immunodeficiency virus (FIV) [160]. In Egypt, HPV was reported [42]; additionally, BPV was documented in bovines [40] and equines [41]. EcPV has not been confirmed in Egypt due to a lack of sufficient data. There are no reports of isolation or cultivation of PVs [161]. It is essential to outline the status of BPVs and EcPVs in Egypt below.

### BPV

BPV is one of the oncoviruses affecting cattle herds, which can lead to reduced hide quality, teat canal obstruction, and significant economic losses [162]. Although cattle are considered the primary reservoir of BPV infection, BPV-1 and BPV-2 have also been investigated in equids, indicating interspecies transmission [163]. Based on phylogenetic analysis, BPVs are classified into four subgroups: *Deltapapillomaviruses* (BPV-1, 2, 13, and 14), *Xipapillomaviruses* (BPV-3, 4, 6, 9, 10, 11, and 12), *Epsilonpapillomaviruses* (BPV-5 and 8), and *Dyoxipapillomavirus* (BPV-7) [164]. The most studied genes in BPVs are those that encode late proteins (L1 and L2) [165].

BPVs have been reported in the Middle East, including Turkey [166], Iraq [164], and Egypt [167]. Common clinical signs of BPV infection include cauliflower-like warts on the skin with epidermal hyperplasia [46]. The location of lesions may differ depending on the virus type; BPV-1 typically appears on the skin of teats and the penis, causing fibropapillomas [168]. BPV-1 and BPV-2 are also associated with skin lesions on the neck, forehead, and back. Additionally, BPV-3 results in warts on the skin, while BPV-4 mainly affects the upper parts of the digestive tract [168].

In Egypt, among all identified types of BPVs, BPV-1 and BPV-2 were only characterized. The first molecular detection and genotyping of circulating BPVs in Egypt were conducted in three governorates, including Giza, Beni Suef, and Menoufia [169]. BPV-2 was first recorded in 2020; sequence analysis revealed a high similarity to reference BPV-2 (*Deltapapillomavirus-4*) strains investigated in Brazil and China [40]. Molecular surveillance, using electron microscopy, of the circulating types of BPVs in four governorates in Egypt, Al-Fayoum, Sohag, Al-Beheira, and Marsa Matrouh, revealed four isolates of *Deltapapillomavirus-4.* Two isolates had a close relationship with an isolate of equine origin [167].

An Egyptian study confirmed that BPV alters hematological parameters, antioxidant balance, collagen content, and γ-catenin content, as well as the levels of trace elements, including copper [170]. BPV-1 is now considered a common cause of equine sarcoids in Egypt [41]. The standard diagnosis of BPVs depends mainly on clinical signs. It is further confirmed by histopathology, IHC analysis, electron microscopy (EM), and molecular identification using PCR, which amplifies the major capsid L1 protein [171]. Besides, PCR has been recommended as a reliable diagnostic tool for the identification and genotyping of BPVs [172].

Diagnostic approaches for BPVs employed in Egypt include clinical signs with gross findings [170], serological identification by ELISA [170], molecular identification via PCR [40], histopathological and IHC characterization [169], evaluation of oxidant-antioxidant biomarkers [170], and transmission electron microscopy (TEM) [40]. PCR was more recommended for the detection of BPVs compared to histopathological and IHC analysis [169].

It is known that cattle have a weak immune response against BPVs, possibly due to the tropism of PVs for epithelia, which results in persistent infection [168], in addition to the virus’s ability to evade the immune system [173]. Autogenous vaccines obtained from warts of infected animals and administered within the same herd were recommended [174]. Additional control measures include strict biosecurity practices, disinfection of farms, vector control, and isolation of infected animals [168]. Otherwise, there is an insufficient level of surveillance and preventive strategies for BPVs in Egypt. Therefore, control of BPV in Egypt requires intensive awareness, continuous screening, improved biosecurity practices, and use of autogenous vaccines formulated from local viral strains.

### EcPV

EcPVs are recognized as the primary cause of cutaneous warts in horses, aural plaques, genital papillomas, squamous cell carcinomas of the penis and prepuce. To date, only seven EcPVs have been investigated in domestic horses [45]. In addition, BPV-1 and BPV-2 are also responsible for equine sarcoids, which are considered the only known interspecies papillomavirus infection [161]. EcPV-1 is thought to cause most cutaneous papillomas to appear on ears, eyelids, and limbs, but EcPV-2 is known to cause genital papillomas scattered as multiple masses on the penis, resulting in discomfort in stallions [46].

In Egypt, previous studies had limited data on EcPVs, while focusing on the presence of BPV-1 as a cause of equine sarcoids without identifying significant phylogenetic variation [41]. The detection of BPV-2 in Egyptian cattle [40] highlights the need for further monitoring to assess its potential impact on equine populations. A recent study in Egypt investigated the presence of equine papillomas in Sharqia province. Among 35 clinical cases of equine neoplasia, only three were confirmed as papilloma, without molecular characterization [175]. Although precise data on EcPVs in Egyptian equine populations are scarce, international studies highlight the importance of these viruses in equine health [176, 177]. Further research is required to determine the prevalence and impact of EcPVs on local horse breeds in Egypt.

## Conclusion

In conclusion, oncogenic viruses continue to pose a persistent threat in Egypt, resulting in significant economic losses and health implications for humans. Furthermore, efforts to improve local breeds through the expansion of live animal and chicken imports, including cattle, sheep, goats, and breeder chickens, have facilitated the spread of oncogenic viruses, particularly those that cause latent infections. To our knowledge, there are no available quantitative estimates about annual economic losses attributed to oncoviruses in poultry or animal sectors in Egypt based on governmental resources and some studies. For underreported viruses in Egypt, such as EcPV, improving surveillance requires better molecular diagnostics, targeted monitoring programs in equine populations, increased awareness among veterinarians and animal owners, and enhanced data reporting through international reporting systems, such as the World Organization for Animal Health (OIE). Really, PCR is a scientific solution for identifying oncogenic viruses due to their biological properties, and specialized labs are also recommended for accurate identification of the causative agent rather than those based on tumor markers. There is still an essential need for continuous monitoring of the current and future situation of these viruses in Egypt.

## Data Availability

No datasets were generated or analysed during the current study.

## Abbreviations

AGID: Agar gel immuno-diffusion test
AGPT: Agar gel precipitation test
ALV: Avian leukosis virus
AMLV: Avian myeloblastosis virus
ASV: Avian sarcoma virus
BAC: Bronchioloalveolar carcinoma
BLV: Bovine leukemia virus
bp: Base pairs
BPV: Bovine papillomavirus
CA: Capsid protein
CAM: Chorioallantoic membrane
CDKs: Cyclin-dependent kinases
CEFs: Chicken embryo fibroblasts
CER: Chicken embryo rough cell line
CFT: Complement fixation test
COFAL: Complement fixation for avian leukosis test
C-onc: Cellular oncogene
CPE: Cytopathic effect
CRFK: Crandell-Rees feline kidney cell line
DEFs: Duck embryo fibroblasts
DNA: Deoxyribonucleic acid
EBL: Enzootic bovine leukosis
EBV: Epstein-Barr virus
EcPV: Equus caballus papillomavirus
ELISA: Enzyme-linked immunosorbent assay
EM: Electron microscopy
env: Envelope gene
FBL: Fetal bovine lung cells
FEFs: Feline embryo fibroblasts
FeLV: Feline leukemia virus
FIV: Feline immunodeficiency virus
FL-74: Feline lymphoblastoid-74 cell line
FPV: Fowl poxvirus
gag: Group-specific antigen gene
GaHV-2: Gallid alphaherpesvirus 2
gL: Glycoprotein L
gp51: Surface glycoprotein-51
gp85: Surface glycoprotein-85
HBV: Hepatitis B virus
HBx: Hepatitis B X-oncoprotein
HCV: Hepatitis C virus
HPV: Human papillomavirus
HTLV-1: Human T-lymphotropic virus type-1
HVT: Turkey herpesvirus
ICP4: Infectious cell protein-4
ICTV: International Committee on the Taxonomy of Viruses
IFA: Immunofluorescence assay
IFAT: Indirect immunofluorescent antibody technique
IHC: Immunohistochemistry
IN: Integrase enzyme
JSRV: Jaagsiekte sheep retrovirus
JSRV-CA: Jaagsiekte sheep retrovirus-capsid protein
LCR: Non-coding long control region
LTRs: Long terminal repeats
MA: Matrix protein
MCPyV: Merkel cell polyomavirus
MDV: Marek’s disease virus
Meq: Marek’s EcoRI-Q oncogene
NC: Nucleocapsid protein
OIE: Office International des Epizooties
OPAV: Ovine pulmonary adenomatosis virus
ORFs: Open reading frames
p53: Protein-53
PCR: Polymerase chain reaction
pol: Polymerase gene
pp38: Phosphoprotein-38
pro: Protease gene
PVs: Papillomaviruses
REV: Reticuloendotheliosis virus
RNA: Ribonucleic acid
RT: Reverse transcriptase enzyme
RT-PCR: Reverse transcriptase PCR
SalHV-2: Salmonid herpesvirus 2
SPF-ECEs: Specific pathogen-free embryonated chicken eggs
SU: Surface protein
tax: Trans-activator X gene
TEM: Transmission electron microscopy
TM: Transmembrane protein
vIL-8: Viral interleukin-8
V-onc: Viral oncogene
vTR: Viral telomerase RNA
WDSV: Walleye dermal sarcoma virus
